# Proteomic and metabolomic profiling of acupuncture for migraine reveals a correlative link via energy metabolism

**DOI:** 10.3389/fnins.2022.1013328

**Published:** 2022-09-29

**Authors:** Lu Liu, Weizheng Li, Linpeng Wang, Pengyun Gong, Tianli Lyu, Dapeng Liu, Yajie Zhang, Yijie Guo, Xiang Liu, Min Tang, Hongke Hu, Chao Liu, Bin Li

**Affiliations:** ^1^Beijing Key Laboratory of Acupuncture Neuromodulation, Department of Acupuncture and Moxibustion, Beijing Hospital of Traditional Chinese Medicine, Capital Medical University, Beijing, China; ^2^School of Engineering Medicine & School of Biological Science and Medical Engineering, Beihang University, Beijing, China; ^3^Key Laboratory of Big Data-Based Precision Medicine, Beihang University, Ministry of Industry and Information Technology, Beijing, China; ^4^Third Affiliated Hospital, Beijing University of Chinese Medicine, Beijing, China; ^5^Shanxi Hospital of Integrated Traditional and Western Medicine, Taiyuan, China

**Keywords:** migraine, proteomics, metabolomics, acupuncture, energy metabolism pathways

## Abstract

Migraine is a neurovascular disease with a high disability rate. Acupuncture treatment has emerged as a safe and viable alternative prophylactic therapy that can effectively alleviate the duration and frequency of migraine attacks. However, the therapeutic mechanisms underlying the effects of acupuncture are yet to be systematically elucidated. In this study, we enrolled female patients with migraine without aura (*n* = 20) and healthy controls (*n* = 10). Patients received acupuncture treatment on DU20, DU24, bilateral GB13, GB8, and GB20, applied three times per week over the course of 4 weeks for 12 sessions in total. Blood samples were collected from the median cubital vein before and after acupuncture treatment. Proteomic and metabolomic profiling was performed using liquid chromatography-mass spectrometry to determine the characteristics of differentially expressed molecules and expression of their corresponding biological pathways as well as to elucidate the pathogenesis of migraine and the biological effects underlying the treatment of migraine with acupuncture. Proteomic and metabolomic profiling of plasma samples from patients with migraine without aura before and after acupuncture treatment revealed enrichment of immune-related pathway functions and the arginine synthesis pathway. Joint pathway analyses revealed significant enrichment of the pentose phosphate and glycolysis/gluconeogenesis pathways in patients with migraine. The glycolysis/gluconeogenesis and riboflavin metabolism pathways were significantly enriched after acupuncture treatment. The expression levels of various key proteins and metabolites, including α-D-glucose, flavin adenine dinucleotide, biliverdin reductase B, and L-glutamate, were significantly differentially expressed before and after acupuncture treatment in patients with migraine without aura. Treatment of migraine with acupuncture was associated with significant changes in key molecules and pathways, indicative of physiological changes in the trigeminovascular system, glutamate neurotoxicity, and other migraine-related physiological changes. Overall, our comprehensive analysis using proteomic and metabolomic profiling demonstrates that energy metabolism may serve as a key correlative link in the occurrence of migraine and the therapeutic effects of acupuncture treatment. Our findings may facilitate the identification of diagnostic and therapeutic modalities in the ongoing search for effective treatments for migraine attacks.

## Introduction

Migraine is a painful chronic neurological disorder characterized by recurrent moderate or severe headaches that typically manifest unilaterally in a pulsating manner (Ashina et al., 2021b). Migraines are partly due to genetic predisposition (Gormley et al., 2016) but can also be aggravated by environmental triggers, leading to nausea, vomiting, and hypersensitivity to light and sound (Headache Classification Committee of the International Headache Society (IHS), 2018). Migraines adversely impact the lives of patients, afflicting approximately 1 billion people regardless of culture and socioeconomic status (GBD 2016 Disease and Injury Incidence and Prevalence Collaborators, 2017; Ashina, 2020). Indeed, migraine is currently the second most burdensome neurological disease worldwide (GBD 2016 Neurology Collaborators, 2019). Migraines impose a heavy economic burden due to both direct and indirect costs. Direct costs include costs incurred by specialist visits, medications, and diagnostic tests, which vary among countries according to different healthcare systems and administration policies. Indirect costs include costs due to lost jobs and reduced productivity, and the economic impact is evident in stressful environments (Agosti, 2018). A large European study reported that 17.7% of men and 28.0% of women lost more than 10 days of activity over a 3-month period due to migraine (Steiner et al., 2014).

Current medications used for migraine treatment include non-steroidal anti-inflammatory drugs, triptans, ergot-type preparations, and calcitonin gene-related peptide (CGRP) receptor antagonists (Ashina et al., 2021a). However, the clinical application of migraine medications is limited due to side effects such as cardiovascular and gastrointestinal issues (Katsarava et al., 2018), as well as their short-term therapeutic effects, which can lead to medication overuse and dissatisfaction with available treatments (Krymchantowski, 2004). Reports suggest that excessive use of antimigraine therapies or analgesics can increase the frequency of headaches (Tfelt-Hansen et al., 2012), while the use of different therapeutic drugs can lead to toxic reactions in the liver, heart, brain, and other organs (Lionetto et al., 2013). Accordingly, there is a critical unmet need to reconsider treatment approaches for the relief and prevention of migraines. As a complementary alternative therapy without severe adverse effects, acupuncture has been reported to exert long-term effects for alleviating migraine recurrence, significantly decreasing the occurrence of days with migraine and migraine attacks, and for reducing pain intensity (Zhao et al., 2017; Xu et al., 2020). A retrospective analysis revealed that acupuncture treatment is cost-effective for individuals with migraine, as the medical costs of patients with migraine and hospitalization of patients with severe migraine can reduce with the use of acupuncture add-on therapy (Tsai et al., 2020). However, the exact pathophysiology of migraine and physiological changes underlying acupuncture treatment of migraine are not fully understood. Previous reports have demonstrated that trigeminovascular system activation, immune effects, and oxidative stress may underscore migraine pathophysiology. Extant research suggests that the onset, progression, and termination of migraine may be associated with neuronal and trigeminovascular pain pathways, and trigeminovascular system activation has been linked to metabolic factors (Goadsby et al., 2017; Charles, 2018). In a rat model of migraine, dysregulation of the mitochondrial dynamic regulatory network was observed in trigeminal neurons, accompanied by suppression of mitochondrial biogenesis and a dynamic shift toward mitochondrial fission (Dong et al., 2017). Mitochondria also interact with the immune system. For instance, the cortical spreading depression (CSD)-induced neuroinflammatory signaling pathway may be associated with activation of the mitochondrial stress-induced mtDNA-triggered NLRP3 inflammasome (Kursun et al., 2021). Insufficient energy in the brain and high levels of oxidative stress are pathophysiological characteristics of patients with migraine (Gross et al., 2019). These findings support the notion that mitochondrial dysfunction is a key factor in migraine pathophysiology.

Given the multifactorial nature of migraines, integrated analysis combining proteomics and metabolomics is crucial for assessing migraine pathophysiology (Lionetto et al., 2013). Proteomics, as a supplement to other “omics” technologies, enables the identification and quantification of overall proteins in biological systems. Technological advances have led to substantial improvements in the quantitative accuracy of mass spectrometry (MS)-based quantitative proteomics, which supports the quantification of more proteins (Liu et al., 2014; Chi et al., 2018). Accordingly, quantitative proteomics holds promising prospects for development and application in the future, including in the discovery of biomarkers and clinical applications. This technology is currently applied in multiple fields and is used for detecting multiple diagnostic biomarkers, revealing pathogenicity mechanisms of disease, screening vaccine candidates for manufacturing, regulating expression patterns of different signals, and interpreting different functional pathways (Suhre et al., 2021). Moreover, metabolomic analyses afford novel perspectives for understanding an organism’s overall health status by evaluating key metabolic changes in living systems. This method reflects changes in genetic modifications, physiological stimuli (e.g., diet and environmental factors), and the gut microbiome to expound on the identification of biomarkers and alterations in biochemical pathways. As such, this method has the potential to promote the development of better strategies for disease prevention and treatment (Kaddurah-Daouk et al., 2008; Wishart, 2019).

To the best of our knowledge, although several studies have performed an “omics” analysis of patients with migraine (Zielman et al., 2016), there is yet to be a study examining the systemic proteomic and metabolomic effects of acupuncture treatment in patients with migraine. In this regard, deeper understanding of the underlying physiological changes will assist in the development of effective treatment strategies. Therefore, the objectives of our study were to elucidate the biological mechanisms of acupuncture treatment in patients with migraine and to identify the differentially expressed molecules and corresponding enriched pathways using proteomic and untargeted metabolic profiling. We hypothesized that several metabolic factors would be associated with acupuncture treatment of migraine. Ultimately, our study aimed to provide more comprehensive and detailed understanding of the association between the molecular status of proteins and metabolites following acupuncture for migraine.

## Materials and methods

### Patients and samples

All patients in our study were enrolled at the Outpatient Department of Acupuncture and Moxibustion, Beijing Hospital of Traditional Chinese Medicine. The study protocol was approved by the Research Ethical Committee of the Beijing Hospital of Traditional Chinese Medicine (ref: 2016BL-081-02). All individuals provided written informed consent for the recruitment for scientific purposes before enrollment. This study was conducted in accordance with the latest version of the Declaration of Helsinki.

Patients with migraine without aura (*n* = 20) who fulfilled the criteria of the International Classification of Headache Disorders (3rd edition, beta version) (Headache Classification Committee of the International Headache Society, 2013) and healthy controls (*n* = 10) were recruited. All the enrolled patients were diagnosed by an experienced neurologist. Patients and controls were matched based on age (20–40 years) and sex (female) to ensure that the groups were as homogenous as possible. Other inclusion criteria included the presence of 2–8 migraine attacks during the 4-week baseline phase, patients with migraine with an initial onset before 50 years of age, and a history of migraine lasting more than 1 year.

The exclusion criteria included other types of primary (e.g., tension-type headache, cluster headache, or chronic migraine) and secondary headaches, history of severe systemic diseases such as immune and nervous system diseases, cardio-metabolic disorders, acute infectious disease, hematopathy, allergies, clinically diagnosed psychiatric disorders, pregnancy, breastfeeding, non-compliance with the baseline headache diary, and the use of any type of prophylactic acupuncture or medication within 3 months before the baseline phase. Subsequently, all patients were instructed to avoid any other analgesics or initiating any other interventions.

Venous blood samples were collected from the median cubital vein in patients with migraine before and after acupuncture treatment (24 h) and in healthy control participants. All samples were drawn using ethylenediaminetetraacetic acid (EDTA)-containing plasma tubes (Shandong Aosaite Medical Devices Co., Ltd., Shandong, China) between 7:30 and 8:30 am. To achieve blinded measurement, the samples were coded. Plasma samples were centrifuged at 1,500 × *g* for 20 min at 4°C and immediately stored at −80°C until the analysis of proteins and metabolites using high-performance liquid chromatography-tandem mass spectrometry (HPLC–MS/MS) was performed. During the 48 h before sampling, participants were instructed to avoid taking analgesics or antimigraine medications, and there were no restrictions on dietary intake.

### Study interventions

The treatments were administered by two licensed acupuncturists with more than 20 years of clinical experience. The acupuncturists received specific training regarding the study purpose, treatment strategies, and quality control (QC). Each patient received 12 acupuncture treatment sessions of 30-min duration, three times per week over 4 weeks. Supplementary Table 1 presents information about the location of acupuncture points and depth of needle insertion.

The acupuncturists performed manual acupuncture at eight acupuncture points, including DU20, DU24, bilateral GB13, bilateral GB8, and bilateral GB20. The therapy involved the use of sterile acupuncture needles (ANDE Needles, Guizhou ANDE Medical Equipment, Co., Ltd., Guizhou, China) with a diameter of 0.25 mm and length of 25–40 mm. After sterilization, acupuncture needles were inserted into acupuncture points and applied with manual manipulation to elicit the “Deqi” sensation. In cases of severe pain [visual analog score (VAS) > 8], ibuprofen (300 mg/capsule; maximal tolerated dose 1,200 mg/day) was used as a rescue medication.

### Assessment of migraine

Detailed headache diaries regarding the duration of migraine diagnosis, number of migraine days, and headache intensity (VAS score) within the 4-week baseline phase and 4-week treatment phase (before and after acupuncture treatment) were obtained from migraineurs. The number of migraine days and average headache intensity were calculated every 4 weeks over an 8-week period. Each patient completed a baseline headache diary for screening and was subsequently interviewed by an experienced neurologist before enrollment.

All patients completed a standardized questionnaire encompassing demographics, headache characteristics, the six-item Headache Impact Test (HIT-6) (Yang et al., 2011), Migraine-Specific Quality-of-Life Questionnaire (MSQ) (Cole et al., 2007), Beck Depression Inventory-II (BDI-II) (Geisser et al., 1997), Beck Anxiety Inventory (BAI) (Leyfer et al., 2006), Montreal Cognitive Assessment (MoCA) (Freitas et al., 2013), and Pittsburgh Sleep Quality Index (PSQI) (Buysse et al., 1989) at baseline and at the end of the 4-week treatment phase. Tables 1, 2 present the relevant demographic and clinical characteristics of the participants.

**TABLE 1:** Demographics and clinical characteristics of healthy controls and migraine patients.

Demographics	Healthy controls (*n* = 10)	Migraine (*n* = 20)	*P*-value^a^
**Age–year**			
Mean (SD)^b^	32.60 (3.95)	32.00 (4.31)	0.7151
Median (IQR)	33.00 (29.00–35.00)	32.00 (30.00–35.25)	
Range	23.00–39.00	26.00–39.00	
**Sex–no. (%)**			
Female^c^	10 (100.00)	20 (100.00)	1.00
**Education–no. (%)**			
Above/below bachelor level^c^	7 (70.00)/3 (30.00)	16 (80.00)/4 (20.00)	0.5416
**Marriage–no. (%)**			
Married/single^c^	9 (90.00)/1 (10.00)	17 (85.00)/3 (15.00)	0.7041
Duration of migraine diagnosis at baseline–year, mean (SD)	NA	13.70 (7.55)	NA
**Accompanying symptoms–no. (%)**			
Nausea or vomiting	NA	17 (85.00)	NA
Photophobia or phonophobia	NA	15 (75.00)	NA
**Use of acute pain medication–no. (%)**	NA	7 (35.00)	NA

^a^All tests were two-sided. Statistical significance was set at *P* < 0.05.

^b^Analyzed using independent samples *t*-test.

^c^Analyzed using Chi-square test. SD, standard deviation; IQR, interquartile range; no. (%), number; NA, not applicable.

**TABLE 2:** Clinical characteristics of 20 migraine patients before and after treatment.

Clinical characteristics	Baseline	After treatment	MD (95% CI)	*P*-value^a^
Days with migraine per 4 weeks, mean (SD)^b,c^	6.10 (5.55)	1.52 (1.66)	4.58 (2.43, 6.73)	0.0003
Mean VAS score, mean (SD)^b^	8.10 (1.62)	4.90 (1.65)	3.20 (1.98, 4.42)	<0.0001
HIT-6, mean (SD)^b^	66.20 (5.02)	57.30 (7.36)	8.90 (5.76, 12.04)	<0.0001
**MSQ, mean (SD)^b^**				
Role restrictive subscale	55.43 (13.45)	77.14 (15.73)	−42.00 (−59.30, −24.69)	0.0001
Role preventive subscale	71.25 (13.56)	85.75 (15.07)	−14.50 (−21.70, −7.30)	0.0005
Emotional subscale	67.67 (22.51)	84.67 (11.87)	−17.00 (−26.58, −7.43)	0.0015
BDI-15 mean (SD)^b^	9.40 (4.99)	4.85 (4.17)	4.55 (2.28, 6.82)	0.0005
BAI, mean (SD)^b^	9.75 (7.49)	4.75 (3.97)	5.00 (2.09, 7.91)	0.0015
MoCA, mean (SD)^b^	27.60 (2.09)	28.15 (1.84)	−0.55 (−1.25, 0.15)	0.1183
PSQI, mean (SD)^b^	5.00 (2.32)	4.50 (2.06)	0.50 (−0.69, 1.69)	0.3905

^a^All tests were two-sided. Statistical significance was set at *P* < 0.05.

^b^Analyzed using paired *t*-test.

^c^Number of days with migraine was defined as the duration of migraine attacks. SD, standard deviation; MD, mean difference; CI, confidence interval; VAS, visual analog scale; HIT-6, six-item Headache Impact Test; MSQ, Migraine-Specific Quality of Life Questionnaire; BDI-II, Beck Depression Inventory-II; BAI, Beck Anxiety Inventory; MoCA, Montreal Cognitive Assessment; PSQI, Pittsburgh Sleep Quality Index.

### Protein extraction and enzymatic solution

Plasma samples were diluted with 8 M urea containing 1% sodium dodecyl sulfate (containing a protease inhibitor) and lysed on ice, and vortex oscillations were performed every 10 min. After centrifugation at 12,000 × *g* for 20 min at 4°C, soluble protein lysates were collected and identified using the bicinchoninic acid protein assay. Then, 100 μg of protein from each sample was transferred into a fresh Eppendorf tube. After addition of 2 μL of 0.5 M tris(2-carboxyethyl)phosphine at 37°C, 4 μL of 1 M iodoacetamide was added, and the mixture was protected from light at room temperature for 40 min. After adding five volumes of prechilled acetone at −20°C, precipitation was conducted overnight. The precipitates were washed with 90% acetone solution and centrifuged at 12,000 × *g* for 20 min at 4°C. After thorough drying at room temperature, the proteins were dissolved in 100 μL of 100 mM tetraethylammonium bromide. Sequence-grade modified trypsin (Promega, Madison, WI, USA) was then added at a mass ratio of 1:50 enzyme/protein overnight at 37°C.

### Liquid chromatography with tandem mass spectrometry analysis for proteomics

Desalination was performed using C18 ZipTip (Millipore, Burlington, MA, USA). A Pierce quantitative colorimetric peptide assay (Thermo Fisher Scientific, Waltham, MA, USA) was used for quantification followed by lyophilization. Subsequently, 30 μL of 0.1% formic acid aqueous solution (solvent A) was added to each sample. The whole system comprised an Orbitrap Exploris 480 mass spectrometer (Thermo Fisher Scientific) coupled to an EASY-nanoLC 1200 system (Thermo Fisher Scientific). Samples (3 μL) (Acclaim PepMap C18, 75 μm × 25 cm) were separated with a 130-min gradient. The column temperature was set at 40°C, with a constant flow rate of 250 nL/min. A mass spectrometer with electrospray at an inlet voltage of 2 kV was used. Using 0.1% formic acid aqueous solution (mobile phase A) and 0.1% formic acid acetonitrile (ACN) solution (mobile phase B), a 130 min separation gradient (0 min, 4% B; 10 min, 6% B; 120 min, 50% B; 121 min, 95% B; 130 min, 95% B) was established. The mass spectrometer was operated in data-independent acquisition (DIA) mode, and one full scan was followed by eight windows. The following MS parameters were used: (1) MS: scan range (m/z) = 350–1,200, resolution = 120,000, automatic gain control (AGC) target = 300%, maximum injection time = 50 ms, and charge states = 2–6; (2) HCD-MS/MS: resolution = 30,000, isolation window = 2, AGC target = 200%, maximum injection time = 50 ms, collision energy = 25, 30, 35; (3) variable isolation windows were used for DIA, and each window overlapped by 1 m/z. The DIA MS data were processed using DIA-NN (v.1.7.12) (Demichev et al., 2020), and the “Generate Spectrum Library” option was set. The workflow of DIA-NN commenced with a peptide-centric approach, and the collection of precursor ions supported automatic generation from a protein sequence database (library-free mode) (Demichev et al., 2020). The FASTA file used a human proteome database containing Swiss-Prot sequences (downloaded from the UniProt database on March 21, 2020; containing 20,365 proteins). A false discovery rate of <0.01 was set.

### Metabolite extraction

For untargeted metabolomic analysis, the stored samples were thawed at 4°C. Then, 400 μL of methanol (MeOH) and 400 μL of ACN were added to a 100 μL plasma sample and centrifuged at 13,000 rpm for 15 min at 4°C. The supernatant was collected and evaporated to dry with a vacuum concentrator. The dry extract was resuspended in a 1:1 ACN:H_2_O solution (100 μL centrifuged at 13,000 rpm for 15 min at 4°C). The supernatant was collected and stored at −80°C.

### Liquid chromatography with tandem mass spectrometry analysis for metabolomics

For untargeted metabolomic analysis in both positive and negative ion mode, the whole system comprised an HPLC–MS/MS on a Triple TOF 6600plus mass spectrometer (AB SCIEX, Foster City, CA, USA) coupled to an Agilent 1290 LC system (Agilent, Palo Alto, CA, USA), while the ACQUITY UPLC BEH Amide column (100 mm × 2.1 mm, 1.7 μm, Waters) was used for LC separation. For positive and negative ion modes, the ion spray voltage was set to 5,000 V (ESI+) and −4,000 V (ESI−), respectively. The MS scanning range was collected between 60 and 1,200 m/z. The curtain, heating, and atomization gas flow was set at 35, 60, and 60 psi, respectively. The injection volume was set at 5 μL, and the gradient separation was 12 min. The flow rate of the column was 500 μL/min, and the column temperature was set to 25°C. Mobile phase A was water (containing 25 mM ammonium acetate and 25 mM ammonia), and mobile phase B was pure ACN. Gradient settings were set at 95% of the initial conditions of mobile phase B for 0.5 min, then the linear gradient shifted from 95 to 65% of mobile phase B in 6.5 min, and then from 65 to 40% in 1 min; 40% of the elasticity of mobile phase B was maintained for 1 min. Subsequently, a 0.1-min gradient was used to return to the initial condition, which lasted for another 2.9 min. In the instrumental analysis, one QC sample was inserted for every seven analytical samples. For untargeted metabolomics analysis, the original data collected by MS were converted using ProteoWizard (version 3.0.6150). The converted file was further processed using XCMS (version 1.46.0) for peak identification and retention time alignment to obtain the peak list. The main parameters were as follows: minimum peak width, 5 s; maximum peak width, 30 s; ppm deviation, 25 ppm; and signal-to-noise threshold (snthresh), 3.

### Statistical analysis

Continuous data are summarized as mean [standard deviation (SD)] and median [interquartile range (IQR)] values, and categorical data are described as numbers (%). The Shapiro–Wilk test was performed to assess normality. Comparisons of two groups (before vs. after treatment/healthy control vs. migraine) were conducted using an independent samples/paired *t*-test or Mann–Whitney *U*-test. The relationship between categorical variables was evaluated using the chi-squared test and Fisher’s exact test. Bonferroni adjustment was applied to control the family wise error rate in these comparisons in consideration of the main hypothesis of the study. In the analysis of demographics and clinical characteristics, Statistica 20.0 software (TIBCO Software Inc, Palo Alto, CA, USA) was used, and the statistical significance level was set at *P* < 0.05.

Missing values were processed using the “Wu kong” platform (Wang et al., 2020). Protein data rows with a missing value ratio of >80% in the quantitative results were deleted. The Seq-KNN method was used to fill in missing values. For quantitative proteins, deviation between samples was reduced using the median normalization method. The *P*-value of the protein was calculated using an unpaired two-sided Welch’s *t*-test, and the fold change was calculated according to the ratio of the two groups. The data were filtered according to a *P*-value of <0.05, and fold change of >1.2 or <1.2^–1^ in volcano plots. The gene names of differentially expressed proteins (DEPs) were drawn using the UniProtKB/Swiss-Prot public database. clusterProfiler (v.3.18.0) in R studio (v.1.2.5033) was used to enrich the Gene Ontology (GO) terms and Kyoto Encyclopedia of Genes and Genomes (KEGG) pathways (Yu et al., 2012).

MetDNA^1^ was used for metabolite identification (Shen et al., 2019). Statistical analyses were performed using the StatTarget package (Luan et al., 2018). The QC-base random forest signal correction algorithm (QC-RFSC) was used to perform signal correction based on QC. We generated a preliminary filter according to a *P*-value of <0.05 and fold change of >1.2 or <1.2^–1^ in volcano plots. Subsequently, the data in the positive and negative ion modes were merged, and multivariable statistical analysis was conducted using SIMCA-P software version 14.1 (Umetrics, Umea, Sweden). An orthogonal partial least squares discriminant analysis (OPLS-DA) model was constructed, and the model was verified by a substitution test of 200 iterations. Differentially expressed metabolites (DEMs) were screened by variable importance in projection of >1, *P*-value of <0.05, and fold change of >1.2 or <1.2^–1^. Metabolic and joint pathway analyses were performed using MetaboAnalyst 5.0^2^ (Pang et al., 2021). Sample normalization by median was selected. Pareto scaling and logarithmic normalization were selected for data scaling and transformation, respectively.

SIMCA-P software was used to construct an OPLS-DA model for multivariate correlation analysis. Data were scaled to unit variance and subjected to linear transformation. After data processing, regression analysis was performed between quantified plasma molecules (proteins and metabolites) and clinical variables of pain intensity (VAS) in patients with migraine who received acupuncture treatment. VAS was used as a single y-variable, and x-variables were molecular quantitative values. In S-plot plots, the abscissa and ordinate coordinates represented the co-correlation and correlation coefficients of the principal component and molecule, respectively. To verify the results obtained using the above method, Spearman correlation analysis was performed, and the results were illustrated as a heatmap.

Machine learning methods provide requisite data parsing, integration and analysis, and binary output capability characteristics, which enables the construction of multiple classification models using combinations of biomarkers. In recent years, machine learning has demonstrated broad application prospects for disease diagnosis, health management, and other medical and health fields. In this study, machine learning analysis was performed using MetaboAnalyst 5.0 (see text footnote 2). The classification was performed using linear support vector machines (SVMs), a supervised machine learning algorithm that efficiently separates datasets by constructing a hyperplane in an N-dimensional space. The mass spectrum intensity values were used as the input of the machine learning model to construct receiver operating characteristic curve analysis and calculate the area under the curve (AUC) to characterize the relationship between specificity and sensitivity.

## Results

### Demographic features and clinical characteristics

We recruited 20 patients with migraine without aura and 10 age- and sex-matched healthy controls to ensure the groups were as homogenous as possible (Table 1). To evaluate the impact of acupuncture on proteomics and metabolomics in patients with migraine without aura, we compared paired samples obtained before and after treatment (*n* = 20). The headache characteristics and comorbidities of the migraineurs before and after acupuncture treatment are presented in Table 2. Table 2 provides mean scores on the BDI-II (mean score 9.40 is indicative of normal, BDI-II score, 0–10), the BAI (mean score 9.75 is indicative of normal, BAI score, 0–14), the MoCA (mean score 27.60 is indicative of normal, MoCA score, 26–30) and the PSQI (mean score 5.00 is indicative of normal, PSQI score, 0–5) at baseline. Co-morbid depression, anxiety, cognitive impairment and sleep disturbance were not found in these migraine patients. Significant changes were observed in the number of migraine days, VAS score, HIT-6 scale, and MSQ scale after acupuncture treatment, which are consistent with the results of our previous studies (Wang et al., 2011; Zhao et al., 2017; Xu et al., 2020; Table 2). Specifically, acupuncture resulted in significantly greater reduction in the number of migraine days after the treatment phase, with a mean difference of 4.58 (95% confidence interval 2.43–6.73; *P* = 0.0003) days compared with that at the baseline phase. A significant decrease in VAS score was observed from baseline to the end of acupuncture treatment (8.10 ± 1.62 vs. 4.90 ± 1.65; *P* < 0.0001). At baseline, the mean HIT-6 score was 66.20, suggesting that headache had a severe impact. The decrease in mean HIT-6 score from baseline was significant at week 4 (*P* < 0.0001), resulting in a mean HIT-6 score at week 4 that was below the severe impact threshold. All subscales of the MSQ scale improved significantly by week 4 relative to baseline. Moreover, in patients with paired samples before and after acupuncture treatment, total scores on the BDI-II and BAI scales were significantly lower at week 4 than at baseline. However, we did not observe any significant differences in the changes in MoCA and PSQI scores from baseline to week 4.

### Proteomic and metabolomic profiling of plasma from patients with migraine

**FIGURE 1:**
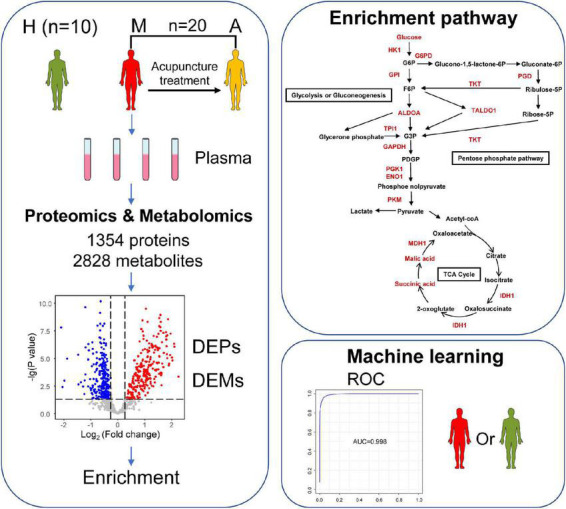
Study design and workflow. Overview patterns of blood sample collection from migraine without aura patients, including M group (migraine patients before acupuncture treatment) (*n* = 20), A group (migraine patients after acupuncture treatment) (*n* = 20), and H group (healthy controls) (*n* = 10). A total of 1,354 proteins and 2,828 metabolites were identified by proteomics and metabolomics. Differentially expressed biomarkers (DEPs and DEMs) are involved in pathways including a variety of immune responses and changes in energy metabolism. This may help us to understand the pathogenesis of migraine and the potential biological effects of acupuncture in the treatment of migraine. Furthermore, this may allow the identification of potential biomarker combinations for the classification of migraine without aura patients and healthy controls by using a machine learning strategy.

We collected 50 plasma samples in total, including 20 blood samples from the same group of migraine patients without aura before and after acupuncture treatment, 20 plasma samples from patients before acupuncture treatment (M group), and 20 plasma samples from patients after acupuncture treatment (A group). In addition, 10 plasma samples of healthy control group (H group) were included. To characterize changes in protein and metabolite expression, we used DIA proteomics and UPLC-MS/MS untargeted metabolomics to analyze the protein and metabolite profiles in patients with migraine after acupuncture treatment (Figure 1). In total, 1,354 proteins (Supplementary Table 2) and 2,828 metabolites (1,389 in negative ion mode and 1,439 in positive ion mode) (Supplementary Tables 3, 4) were identified and quantified. For untargeted metabolomics, total ion chromatograms (TIC) of QC samples under positive and negative ion modes (Supplementary Figure 1) revealed good overlap between the retention time and response intensity of each chromatographic peak. Furthermore, the violin plot of the results of median normalization for proteomic and metabolomic data demonstrated that the biases between samples decreased (Supplementary Figure 2). Collectively, these results highlight the stability and reproducibility of the data.

### Differentially expressed protein and differentially expressed metabolite expression and functional enrichment between patients with migraine and healthy controls

Differentially expressed proteins were screened according to the criteria of fold change and *P*-value (Supplementary Figure 3). The volcano plots and OPLS-DA model were further used to screen DEMs according to the combination of *P*-value of <0.05, fold change of >1.2 or <1.2^–1^ (Supplementary Figure 3), and variable importance in projection value of >1. The permutation test with 200 iterations (Supplementary Figure 4) and high R2X, R2Y, and Q2 confirmed the good quality of each supervised model.

**FIGURE 2:**
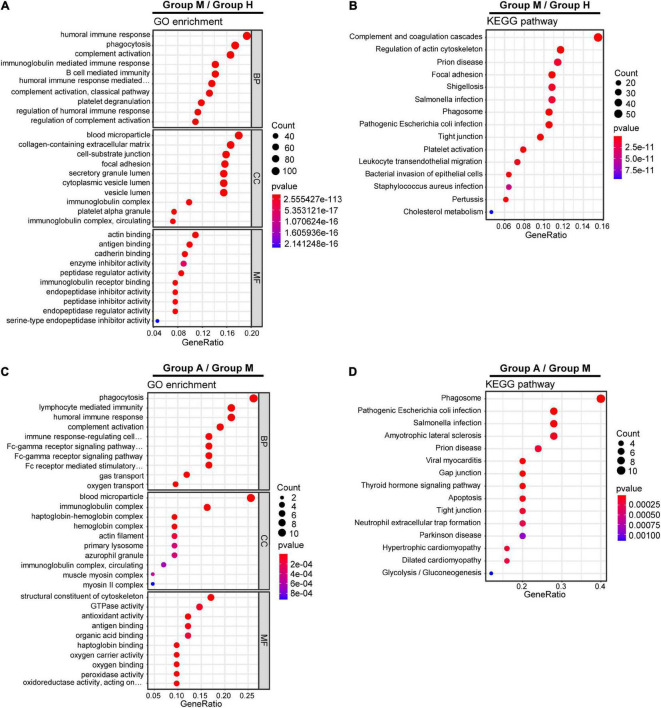
Gene Ontology (GO) terms and KEGG pathways enrichment analysis of DEPs. GO enrichment analysis **(A)** and KEGG pathway analysis **(B)** of DEPs in M group and H group. GO enrichment analysis **(C)** and KEGG pathway analysis **(D)** of DEPs in M group and A group.

**FIGURE 3:**
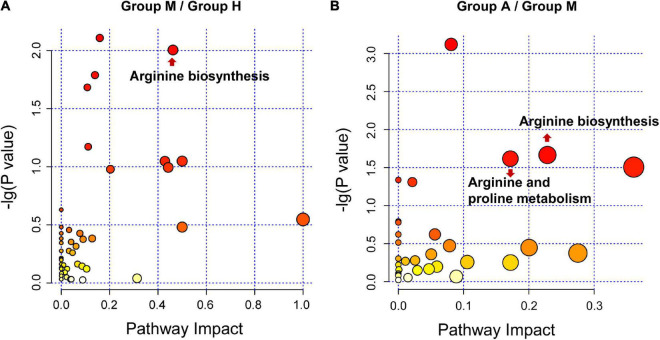
Pathway analysis for DEMs. **(A)** The metabolic pathway analysis between H group and M group. **(B)** The metabolic pathway analysis between M group and A group. The size and color of each bubble is based on the pathway impact value and *P*-value, respectively.

We compared the proteomic changes in patients with migraine without aura and identified 526 DEPs that centered on the involvement of the humoral immune response and complement and coagulation cascades (Figures 2A,B) between the M and H groups. In addition, we identified 114 DEMs (59 in negative ion mode and 55 in positive ion mode) in the comparison of the M and H groups. The related metabolic pathways (Supplementary Table 5) predominantly involved arginine biosynthesis (Figure 3A). Joint pathway analysis (Supplementary Table 6) revealed significant enrichment of the pentose phosphate and glycolysis/gluconeogenesis pathways (Supplementary Figure 5A).

**FIGURE 4:**
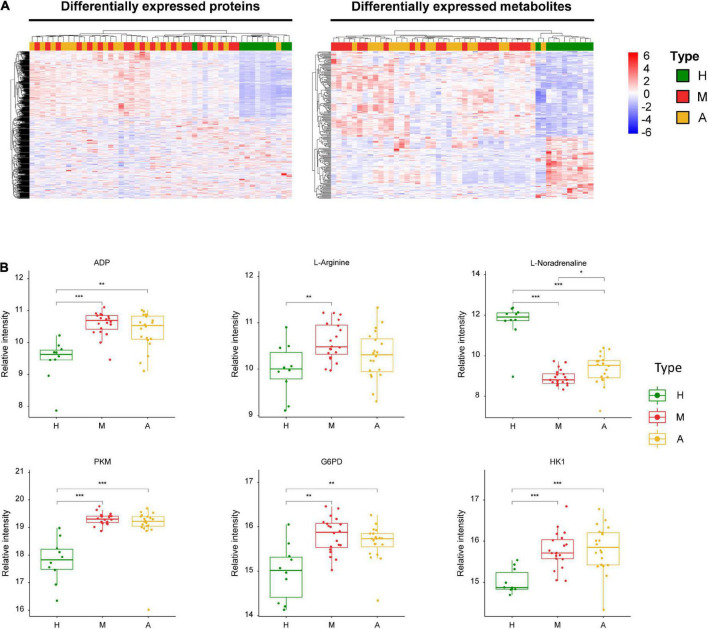
Dysregulated proteins and metabolites in H group and M group. **(A)** Heatmap of DEPs and DEMs between H group and M group. **(B)** The expression level change of the key proteins and metabolites with significant difference between H group and M group. Asterisks indicate statistical significance based on unpaired two-sided Welch’s *t*-test. Significance levels: *P < 0.05, **P < 0.01, ***P < 0.001.

Our analysis also revealed three energy metabolism-related enzymes, including pyruvate kinase (PKM), glucose-6-phosphate dehydrogenase (G6PD), and hexokinase 1 (HK1) (Figure 4). In addition to these proteins, we identified key dysregulated metabolites, including L-arginine, ADP, and L-noradrenaline. These changes in key molecules, especially α-D-glucose and ADP, were indicative of energy metabolism disorders and oxidative stress in patients with migraine.

### Differentially expressed protein and differentially expressed metabolite expression and functional enrichment after acupuncture treatment for patients with migraine

We identified 29 DEPs between groups A and M. GO and KEGG pathway enrichment analyses revealed that DEPs were highly enriched in immune response and complement activation (Figures 2C,D). Further, we identified 69 DEMs (45 in negative ion mode and 24 in positive ion mode) in the comparison of groups A and M. DEMs were uploaded to MetaboAnalyst, and the pathway impact value and *P*-value were calculated to evaluate the importance of the affected metabolomic pathways. Among these altered pathways (Supplementary Table 7), arginine biosynthesis, arginine metabolism, and proline metabolism were notable pathways of interest (Figure 3B). To integrate the DEPs and DEMs in the pathway, we conducted a joint pathway analysis (Supplementary Table 8). We observed that the DEPs and DEMs were significantly enriched in riboflavin metabolism, arginine biosynthesis, and glycolysis/gluconeogenesis pathways (Supplementary Figure 5B).

**FIGURE 5:**
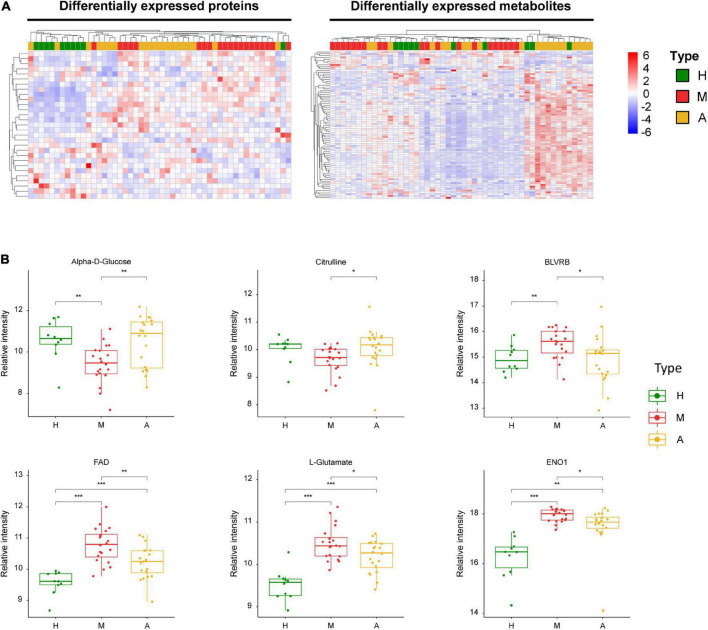
Dysregulated proteins and metabolites between M group and A group. **(A)** Heatmap of DEPs and DEMs between M group and A group. **(B)** The expression level change of the key proteins and metabolites with significant difference between M group and A group. Asterisks indicate statistical significance based on unpaired two-sided Welch’s *t*-test. Significance levels: *P < 0.05, **P < 0.01, ***P < 0.001.

Figure 5 summarizes the key dysregulated molecules, including alpha-D-glucose, flavin adenine dinucleotide (FAD), L-glutamate, biliverdin reductase B (BLVRB), citrulline, and enolase 1 (ENO1). We evaluated the correlation between these key dysregulated molecules and pain intensity based on VAS. We observed multivariable correlations of these key molecules with pain intensity in the OPLS-DA model. Among the key molecules, alpha-D-glucose, citrulline, BLVRB, FAD, and L-noradrenaline exhibited a significant correlation with pain intensity, and VAS was positively associated with BLVRB and FAD and negatively related with alpha-D-glucose, citrulline, and L-noradrenaline (Supplementary Figure 6).

### Identification of patients with migraine using machine learning

**FIGURE 6:**
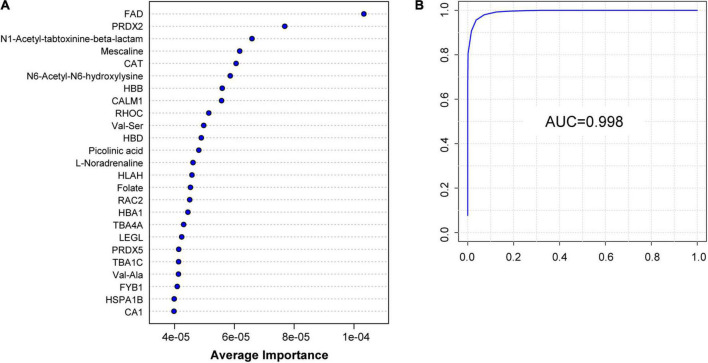
Identification of H group and M group by machine learning of proteomic and metabolomic features. **(A)** Top 25 characteristic molecules prioritized by SVM model by the average importance. **(B)** Receiver operating characteristic (ROC) of the SVM model can classify H group and M group perfectly; AUC: 0.998.

To further explore potential diagnostic biomarkers to distinguish migraine cases from controls, we adopted a machine learning approach using SVMs based on DEPs and DEMs screened from proteomic and metabolomic data. The model achieved a high AUC for classification based only on the first 25 variables, providing sufficient estimates of the AUC results of the first 5–100 biomarkers (Supplementary Figure 7). The 25 key biomarkers, including 16 proteins and 9 metabolites, were ranked by average importance (Figure 6A). The model achieved an AUC of 0.998 (Figure 6B) in our dataset. Receiver operating characteristic curve analysis was performed to quantify the diagnostic performance of each of the 25 previously highlighted individual plasma metabolites. Notably, FAD was a highly predictive metabolite identified by OPLS-DA in the dataset and exhibited the highest average importance in the construction of the SVM model.

## Discussion

### Energy metabolism

Migraines may be interpreted as an adaptive and conservative behavioral response to an imbalance between the supply of and demand for brain energy. In recent years, advanced high-throughput techniques have increasingly been applied to different aspects of brain metabolism in migraineurs, and the metabolic mechanisms underlying migraine pathophysiology have partially been elucidated in animal studies (Gross et al., 2019). The recovery of brain energy and reduction of oxidative stress are thought to be facilitated by the onset of the migraine itself (Borkum, 2018, 2021). Moreover, growing clinical evidence has implicated an insufficient amount of brain energy or limited antioxidant capacity in migraine attacks (Aytaç et al., 2014). The application of ^31^P-magnetic resonance spectroscopy (^31^P-MRS) has revealed that mitochondrial oxidative phosphorylation in the brains of migraineurs is impaired during both ictal (Welch et al., 1989) and interictal periods (Montagna et al., 1994; Kim et al., 2010; Reyngoudt et al., 2012). Numerous reports based on ^31^P-MRS and morphological, biochemical, and genetic studies (Stuart and Griffiths, 2012) have indicated that an energy supply imbalance (i.e., insufficient energy generation coupled with increased consumption) occurs in patients with migraines. These findings suggest that when the energy demand exceeds a threshold level, the metabolic prerequisites for a migraine may be attained, which triggers the occurrence of migraines. Riboflavin and coenzyme Q10 are key components in the regulation of the mitochondrial respiratory chain and are recommended as effective prophylactic treatments for migraines (Holland et al., 2012; Pringsheim et al., 2012). These notable findings suggest that improved energy metabolism may reduce migraine susceptibility. In this study, we compared patients with migraine and healthy individuals, as well as patients with migraine before and after acupuncture treatments. Our results indicated that acupuncture treatment for migraine without aura reversed the levels of key molecules and stimulated systematic changes in immune-, arginine-, and energy metabolism-related pathways based on proteomic and metabolomic data. Further, we established a correlation between pain intensity and key molecular changes in patients with migraine. Collectively, our analyses of associated pathways and corresponding molecular changes related to energy metabolism provide evidence for the influence of energy metabolism on migraines.

We detected three energy metabolism-related enzymes (PKM, G6PD, and HK1) among the DEPs in the healthy controls and patients with migraine (Figure 4B). These enzymes are primarily involved in pentose phosphate and glycolysis/gluconeogenesis pathways (Supplementary Figure 5A). Via the catalysis of PKM in the glycolytic pathway reaction, a phosphate group is transferred from phosphoenolpyruvate to ADP, resulting in the production of ATP (Wang et al., 2018). The rate-limiting enzyme G6PD generates cytosolic NADPH in the oxidative pentose phosphate pathway for use in biosynthesis and oxidative defense (Zhong et al., 2021). HK1 phosphorylates glucose by consuming extracellular ATP. As the major contributors to brain energy metabolism (glucose metabolism), these enzymes that are implicated in ATP synthesis and glucose catabolism play a crucial role in migraine pathophysiology (Gross et al., 2019); therefore, the importance of these enzymes in energy metabolism-related pathways cannot be ignored. Several medications for acute migraine, such as corticosteroids, induce gluconeogenesis and are effective for treating refractory migraines and status migrainosus (Woldeamanuel et al., 2015). In hypoglycemia, cortisol, adrenaline, and noradrenaline protect cells by inhibiting insulin activity, inducing protein catabolism, and enhancing gluconeogenesis and glycogenolysis. Glycolysis and pentose phosphate pathways involved in the biological effects of changes in patients with migraine and healthy individuals are key clues to changes in energy metabolism, in which the response of these three key proteins perform to be instrumental and can serve as key evidence for changes in energy metabolism.

We also observed lower levels of alpha-D-glucose (Figure 5B) and higher levels of ADP (Figure 4B) in patients with migraine compared than in healthy controls. The aforementioned impairment in oxidative phosphorylation manifests as decreased organic phosphate levels and phosphorylation potential, higher ADP levels, and glucose hypometabolism (Welch et al., 1989; Kim et al., 2010), all of which lead to energy metabolism dysregulation, which is consistent with our data. There is also evidence of impaired oxidative phosphorylation in the brains of migraineurs during the ictal (Welch et al., 1989) and interictal periods (Montagna et al., 1994; Kim et al., 2010; Reyngoudt et al., 2012).

Previous studies have suggested that patients with migraines have insufficient metabolic reserves to satisfy the cerebral high energy demands (Lisicki et al., 2018). This causes these individuals to be susceptible to perturbations in cortical homeostasis. Therefore, it is conceivable that acupuncture treatment, in addition to riboflavin, may be beneficial for migraineurs with impaired energy metabolism, since acupuncture may provide an alternative source of fuel for the brain. Further clinical and animal studies are needed to validate the effects of acupuncture on energy metabolism in patients with migraine. Acupuncture may increase antioxidant levels, decrease oxidative stress, and downregulate neuronal reactivity, thereby reducing the energy demands of the brain, increasing mitochondrial biogenesis, and restoring brain energy homeostasis. Our results suggest that energy metabolism regulation underscores the effects of acupuncture in patients with migraine, which is consistent with the proteomic and metabolomic findings in the healthy controls and patients with migraine.

The comparison of patients with migraine before and after acupuncture treatments revealed several key molecules, including L-arginine (Figure 4B) and alpha-D-glucose (Figure 5B). However, no significant differences were observed in the levels of these molecules between the acupuncture and healthy groups (*P* > 0.05). Conversely, we observed that the levels of several key molecules, including alpha-D-glucose, FAD, L-glutamate, citrulline, ENO1, and BLVRB, were significantly altered after acupuncture treatment compared with pre-treatment values (Figure 5B) (*P* < 0.05). ENO1, which is a metabolic enzyme involved in pyruvate synthesis, is a key enzyme in glycolysis. As a plasminogen receptor, ENO1 activates plasmin and degrades the extracellular matrix. Based on current characterization of the biochemical and immunological features of ENO1, it may affect the induction of strong cellular immune and specific humoral responses.

Our joint pathway analysis revealed that changes occurred in riboflavin metabolism and glycolysis pathways after acupuncture compared with pre-treatment values. In accordance with the evidence-based American Academy of Neurology guidelines (Holland et al., 2012), riboflavin is recommended as a Level B medication for migraines in adults, and its effectiveness for migraine prevention has been reported in many clinical studies (Schoenen et al., 1998; Boehnke et al., 2004; Thompson and Saluja, 2017). The properties of riboflavin are also reflected in its neuroprotective functions, which include improvements in neuroinflammation, glutamate excitotoxicity, mitochondrial dysfunction, and oxidative stress. In various neurological diseases, such as migraines, multiple sclerosis, and Parkinson’s disease, the neuroprotective effects of riboflavin may play a key role in multiple pathways (e.g., iron metabolism, mitochondrial function, myelin formation, and antioxidation) that may be impaired at the physiological level. Riboflavin is involved in the oxidized glutathione recycling process and the metabolism of fats, proteins, and carbohydrates. Further, it plays a crucial role in the mitochondrial electron transport chain (Bütün et al., 2015; Marashly and Bohlega, 2017), however, a point that cannot be ignored is the excessive use of riboflavin may induce compensatory downregulation of riboflavin dependent enzymes. These changes in biological effects before and after acupuncture treatment can undoubtedly be used as evidence of changes in energy metabolism. These changes themselves may be crisscrossing and complex, but when we relate them to the corresponding molecular changes and consider the correlative intrinsic interrelationships, the role of changes in energy metabolism emerges.

Biliverdin reductase B family members are general flavin reductases that play a key role in the maintenance of cellular redox. BLVRB is thought to be capable of dictating cell fate alone (Duff et al., 2020). The BLVRB class of enzymes catalyzes the NADPH-dependent reduction of multiple flavin substrates and has emerged as a critical player in cellular redox regulation (Redzic et al., 2021). In the current study, FAD was identified as the molecule with the highest average importance in the SVM classification model that distinguished patients with migraine from healthy controls. FAD expression is upregulated in migraine patients, which may be due to the presence of reductive block in hypoxia and inflammatory signaling. Meanwhile, the level of FAD decreased after acupuncture treatment. These findings suggest alterations in the electron transport chain, which could impact ATP synthesis and energy metabolism. FAD is an essential coenzyme form of riboflavin, and plasma FAD levels have been reported to be consistent in healthy individuals who were administered low doses of riboflavin (Hustad et al., 2002). In humans, mitochondria also produce FAD, which plays a key role in mitochondrial energy metabolism (Mosegaard et al., 2020). These results highlight potential energy metabolism disorders and imbalance in energy regulation in patients with migraine.

### Arginine metabolism

Arginine is an essential amino acid that regulates blood flow by modulating vascular endothelial cells. An increase in serum L-arginine level during the migraine interictal period has been reported (Reyhani et al., 2017). Clinical evidence suggests that nitric oxide (NO) also plays a key role in migraines. NO is synthesized from arginine via endothelial nitric oxide synthase (NOS). Studies focusing on substances that release NO and trigger migraines have provided evidence for the importance of NO in migraine pathogenesis (Demartini et al., 2019). In patients with migraines, endothelial dysfunction and oxidative stress have been linked to L-arginine/NO system dysfunction (Erdélyi-Bótor et al., 2017). The present metabolomics analysis identified changes in the arginine synthesis pathway in patients with migraine compared with those in healthy controls (Figure 3A), as well as in patients with migraine before and after acupuncture treatment (Figure 3B). Our data also revealed the presence of arginine biosynthesis dysregulation, including dysregulation of L-arginine, L-glutamate, citrulline, and L-aspartate.

The glutamate excitotoxicity hypothesis suggests that excessive glutamate damages neurons by causing dysfunction and degeneration (Lau and Tymianski, 2010). Glutamate is a critical neurotransmitter in the pathophysiology of migraine headaches and central sensitization due to its excitatory action on nociceptive neurons in the trigeminovascular system (Hoffmann and Charles, 2018). Our data revealed increased L-glutamate levels in patients with migraines (Figure 5B). Elevated glutamate levels in blood samples have been reported in migraineurs during both ictal and interictal periods (Ferrari et al., 1990; Martínez et al., 1993; Campos et al., 2013). Notably, in the present study, we observed a decrease in glutamate levels after acupuncture treatment. Effective preventive treatments for migraine that have distinct mechanisms of action have been reported to significantly lower plasma glutamate levels (Ferrari et al., 2009).

Citrulline, which is a precursor of NO synthesis, was downregulated in migraine patients (Figure 5B). This could be at least partly because migraine attacks were caused by enhanced NO production via the NOS-induced conversion of arginine to citrulline and NO, resulting in the high-output NO synthesis pathway (Gruber et al., 2010). NO may participate in the mechanisms underlying migraines by triggering neurogenic inflammation and activation of fibers conveying nociceptive inputs to the trigeminal ganglion. Citrulline has been reported to remain at low levels in other types of primary headaches, which is consistent with our data (D’Andrea et al., 2019). While citrulline was upregulated after acupuncture treatment, rodent studies in which citrulline attenuated the propagation velocity of KCl-induced CSD support the notion that increased citrulline levels alleviate migraines (Kurauchi et al., 2017).

High NO levels induce CGRP expression and activate the trigeminovascular system. In this regard, NO may modulate mitochondrial function via various mechanisms (Ghasemi et al., 2018). NO-mediated suppression of the mitochondrial electron transport chain may constitute an effect of NO on mitochondrial activity (Bolanos et al., 1994). Additionally, alterations in platelet activity may play a critical role in migraine pathophysiology through mechanisms that involve the NO pathway (Paolucci et al., 2021). Consequently, the presence of NO in platelets has been proposed as a promising tool for studying changes in NO during migraines.

### Molecular insights into the pathogenesis of migraines: Link between altered bioenergetics and trigeminovascular activation

Factors that trigger migraine may be attributable to dysfunction in brain energy metabolism, hormones, and environmental factors. As a multifactorial disorder, individual differences exist in the primary pathophysiology of migraines. Increasing evidence indicates that migraines may constitute a disorder of brain energetics. The pathophysiology of migraines was the major focus of this study, and we identified the role of energy metabolism and mitochondrial function in migraine pathogenesis.

Extensive evidence suggests that individuals with migraines exhibit abnormalities in energy metabolism in the brain. An increase in sensory stimulation-induced brain reactivity was observed in patients with migraine in almost every sensory modality (de Tommaso et al., 2014), indicating that migraineurs have an imbalance in energy supply demand in the brain, characterized by an increase in demand and decrease in supply. Increased brain energy deficits and/or oxidative stress are also linked to a lower threshold for sensory pain. ATP-sensitive potassium channels, which are widely expressed in the trigeminovascular system, are involved in the relationship between trigeminovascular activation and metabolic stress and are influenced by the intracellular ATP/ADP ratio as well as by cAMP and cGMP pathways (Al-Karagholi et al., 2017). In a study examining the effects of insulin, glucagon, and leptin in a rat model of migraine, changes in the transmission of trigeminal nociceptive inputs were identified, which provides insight into the dysregulated glucose state associated with migraines (Martins-Oliveira et al., 2017). As mentioned above, the nature of blood glucose disorders in migraines is complex and should form the focus of future research efforts.

Collectively, these findings indicate that oxidative stress and energy metabolism play key roles in triggering or exacerbating migraines. Indeed, abnormalities in multiple metabolic pathways in migraines are well established. Disruption of brain energy homeostasis in the trigeminovascular system is also noteworthy, as it may contribute to the pathological changes underpinning migraines. A common pathway for the activation of trigeminal nociceptors may be related to its limbic connections, primarily via the activation of brainstem chemosensitive neurons or direct stimulation of the release of sensory neuropeptides (e.g., pituitary adenylyl cyclase-activating polypeptide and CGRP) by activated meningeal afferent fibers. Conversely, chemosensitive neurons in the diencephalic region and brainstem (Lisicki et al., 2018) may be capable of sensing metabolic changes and inducing the sensitization of the trigeminovascular system. Factors associated with enhanced oxidative stress may also act as contributors to migraine pain (Benemei et al., 2014; Kozai et al., 2014).

Glycogen reserves in the human brain are limited for various reasons, including the presence of the blood–brain barrier (BBB), which excludes the entry of large, energy-dense molecules. In addition, the brain has high energy requirements and is highly dependent on circulating energy sources. The insulin-independent glucose transporter type 1 plays a key role in the transport of glucose in astrocytes, oligodendrocytes, and endothelial cells in the BBB. Notably, the physiological increase in the levels of brain lactate induced by stimulation and the theory of the astrocyte-to-neuron lactate shuttle (ANLS) provide a highly plausible explanation in this regard (Magistretti and Pellerin, 1999). Glucose is the only carbon source of energy for metabolism in the brain and is transported into brain cells across the BBB. As such, brain tissue is highly sensitive to hypoglycemia. The ANLS theory suggests that astrocytes convert blood glucose into lactate during neuronal activation and play a pivotal role in neuronal energy metabolism, including in retinal ganglion cells (Mori et al., 2020). These results and evidence of diminished migraine energy reserves serve as the basis for our understanding of the role of glucose fluctuations and metabolic abnormalities in migraine pathophysiology.

**FIGURE 7:**
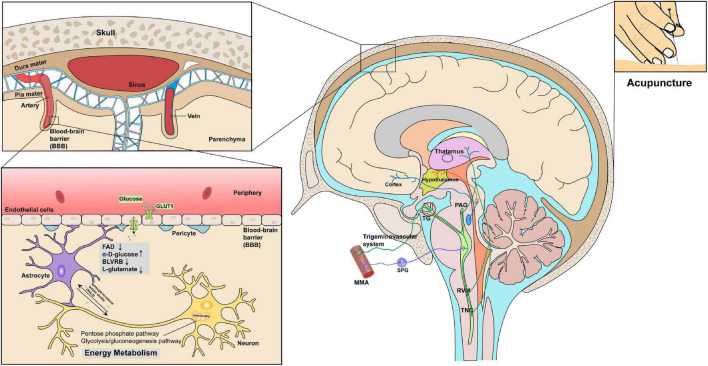
Key proteins and metabolites profiling in migraine patients after acupuncture treatment could reveal the correlative link between acupuncture and energy metabolism in the context of trigeminovascular system. Proteomics and metabolomics profiles indicated that acupuncture treatments may decrease oxidative stress levels (FAD, BLVRB, L-glutamate) while increasing glucose availability (α-D-glucose), thereby aiding in restoring energy homeostasis.

In addition to their effects on brain excitability and reactivity, most preventive migraine therapies have the potential to improve metabolic function (Yang et al., 2014; Gross et al., 2019). Our data shed light on changes in proteomics and metabolomics profiles of the plasma of patients with migraine after acupuncture treatment. Our data reveal a correlative link between acupuncture treatment of migraine and energy metabolism (Figure 7). We hypothesize that acupuncture treatments may decrease oxidative stress levels while increasing glucose availability in the brain, thereby facilitating the restoration of energy homeostasis (Figure 7). In addition, increased mitochondrial biogenesis, reduced excitatory synaptic transmission in pain and inflammatory pathways, and increased antioxidant capacity are possible additional effects of acupuncture on migraine pathophysiology and treatment (Qu et al., 2020; Zhao et al., 2020). Nevertheless, more research is warranted to confirm these potential effects and clinical outcomes.

### Limitations and prospects

Despite the need for a larger sample size, the findings described in this study indicate the existence of several promising biomarker candidates. Our results suggest that acupuncture treatments may be beneficial for patients with migraines by enhancing brain energetics; however, further research (e.g., via MRS and 18F-fluorodeoxyglucose PET) on brain metabolism before and after acupuncture treatment is warranted to draw definite conclusions. Moreover, the impact of placebo acupuncture on proteomic/metabolomic profiles should be evaluated in future studies, as this would enhance the power of the correlations identified herein. Given that sampling from the median cubital vein is more convenient for patients with migraine, we collected plasma samples before and after acupuncture treatment. In this regard, migraine-generating tissues (e.g., meningeal, trigeminal nerve, or CSF) should also be collected to confirm the correlative link between biological differences in disease-specific proteomic/metabolomic profiles and acupuncture treatment for migraine.

Future investigations should harness combined evaluation of brain and energy metabolism with sensory information processing to elucidate the imbalance between brain activity and energy metabolism in patients with migraines. Similarly, studies investigating the role of specific alterations in energy metabolism for other migraine subgroups, such as chronic migraines and migraines with aura, are necessary. Moreover, additional clinical data are required to confirm whether acupuncture treatments that ameliorate dysregulation of energy metabolism result in changes in brain energy usability and sensory information processing.

## Conclusion

Our study provides a comprehensive proteomic and metabolomic analysis of plasma samples from patients with migraines before and after acupuncture treatments and a group of healthy control participants. We analyzed key protein and metabolite changes to characterize the underlying molecular and physiological effects and the potential of acupuncture to treat migraines. Our results indicate that energy metabolism pathways may provide a key link in the association between the development of migraines and the molecular changes underscoring acupuncture treatments. Significant changes in key molecules such as FAD, L-noradrenaline, α-D-glucose, BLVRB, and L-glutamate and their corresponding pathways after acupuncture treatment suggest that physiological changes may involve glutamate neurotoxicity, alongside alterations in the trigeminovascular system and other migraine-related biological effects. Migraine triggers are generally thought to be related to energy imbalances, oxidative stress, and various forms of metabolic dysregulation, including glucose metabolism disorders. Indeed, our conclusions are consistent with these hypotheses. Our data offer an overview of molecular changes in the blood that are induced by activation of the trigeminovascular pain pathway. This information may catalyze future investigations on diagnostic and therapeutic modalities in the ongoing effort to identify effective treatments for migraine attacks.

## Data availability statement

The mass spectrometry data were deposited to the ProteomeXchange Consortium (http://proteomecentral.proteomexchange.org) via the iProX partner repository (Ma et al., 2019) with the dataset identifier: PXD028441.

## Ethics statement

The studies involving human participants were reviewed and approved by the Research Ethical Committee of Beijing Hospital of Traditional Chinese Medicine. The patients/participants provided their written informed consent to participate in this study.

## Author contributions

CL and BL conceived and designed the study, revised the manuscript, and provided key intellectual content. LL, DL, and YZ were responsible for sample collection. WL and LL performed the statistical analyses. LL, WL, and PG wrote the manuscript and prepared the figures and tables. TL, PG, XL, YG, MT, and HH contributed to a thorough revision of the manuscript. All authors read and approved the final manuscript.

## Footnotes

1
http://metdna.zhulab.cn/

2
https://www.metaboanalyst.ca/

## Abbreviations

CSD: cortical spreading depression
MS: mass spectrometry
EDTA: ethylenediaminetetraacetic acid
DIA: data-independent acquisition
AGC: automatic gain control
MeOH: methanol
ACN: acetonitrile
QC: quality control
DEPs: differentially expressed proteins
DEMs: differentially expressed metabolites
OPLS-DA: orthogonal partial least squares discriminant analysis
SVM: support vector machine
TIC: total ion chromatograms
PKM: pyruvate kinase
G6PD: glucose-6-phosphate dehydrogenase
HK1: hexokinase 1
FAD: flavin adenine dinucleotide
ENO1: enolase 1
BLVRB: biliverdin reductase B
NO: nitric oxide
NOS: nitric oxide synthase
CGRP: calcitonin gene-related peptide
BBB: blood–brain barrier
ANLS: astrocyte-to-neuron lactate shuttle.
