# Supplementary material for: Proteomic and metabolomic profiling of acupuncture for migraine reveals a correlative link via energy metabolism
Source: Front Neurosci. 2022 Sep 29;16:1013328. doi: 10.3389/fnins.2022.1013328 (PMC9557737; doi:10.3389/fnins.2022.1013328)
Supplement: Supplementary file 1 [file Data_Sheet_1.docx]

Supplementary Material

## Supplementary Figures

**Supplementary Figure 1.** TIC analysis of QC data for metabolomics. (**A)** The response intensity and retention time of each chromatographic peak under positive ion detection modes. (**B)** The response intensity and retention time of each chromatographic peak under negative ion detection modes.

**Supplementary Figure 2.** Violin plot of before and after median normalization of proteomic and metabolomic data. (**A)** Violin plot of initial proteomic data. (**B)** Violin plot of proteomic data after median normalization. (**C) and (E)** Violin plot of initial metabolomic data under positive and negative ion mode. (**D) and (F)** Violin plot of metabolomic data after median normalization under positive and negative ion mode.

**Supplementary Figure 3.** DEPs and DEMs in different patient groups. Volcano plots for the proteomics and metabolomics data.

**Supplementary Figure 4.** Score plots and permutation test of OPLS-DA for metabolomics. (**A)** OPLS-DA score plot between H group and M group (R2X = 0.446, R2Y = 0.987, Q2 = 0.922). (**B)** Permutation test between H group and M group (R2 = (0.0, 0.886), Q2 = (0.0, -0.436)). (**C)** OPLS-DA score plot between M group and A group (R2X = 0.446, R2Y = 0.987, Q2 = 0.922). (**D)** Permutation test between M group and A group (R2 = (0.0, 0.948), Q2 = (0.0, -0.494)).

**Supplementary Figure 5.** The joint pathway analysis of proteomics and metabolomics. (**A)** The joint pathway analysis between H group and M group. (**B)** The joint pathway analysis between M group and A group. The size and color of each bubble is based on pathway impact value and *P* value, respectively.

**Supplementary Figure 6.** Correlation analysis of Dysregulated molecules and VAS. (A) S-plot obtained from OPLS-DA regression model (R2X=0.221, R2Y=0.569, Q2=0.43). (B) Spearman's correlation heatmap. Red and blue represents positive and negative correlations, respectively. * *P* < 0.05, ** *P* < 0.01, *** *P* < 0.001.

**Supplementary Figure 7.** ROC curve analysis of different marker combinations and prediction accuracy. (**A)** ROC curves of 6 biomarker combination models. (**B)** The prediction accuracy of each model increases gradually with the increase of features.

## Supplementary Tables

**Supplementary Table 1.** Acupoints used in the acupuncture treatment

**Supplementary Table 2.** The proteomic quantitative data matrix for the samples

**Supplementary Table 3.** Metabolomic (negative ion mode) quantitative data matrix for the samples

**Supplementary Table 4.** Metabolomic (positive ion mode) quantitative data matrix for the samples

**Supplementary Table 5.** Detailed information on the metabolic pathway analysis between the H and M groups

**Supplementary Table 6.** Detailed information on the joint pathway analysis between the H and M groups

**Supplementary Table 7.** Detailed information on the metabolic pathway analysis between the M and A groups

**Supplementary Table 8.** Detailed information on the joint pathway analysis between the M and A groups
