# Supplementary figures and images for: Proteomic and metabolomic profiling of acupuncture for migraine reveals a correlative link via energy metabolism
Source: Front Neurosci. 2022 Sep 29;16:1013328. doi: 10.3389/fnins.2022.1013328 (PMC9557737; doi:10.3389/fnins.2022.1013328)

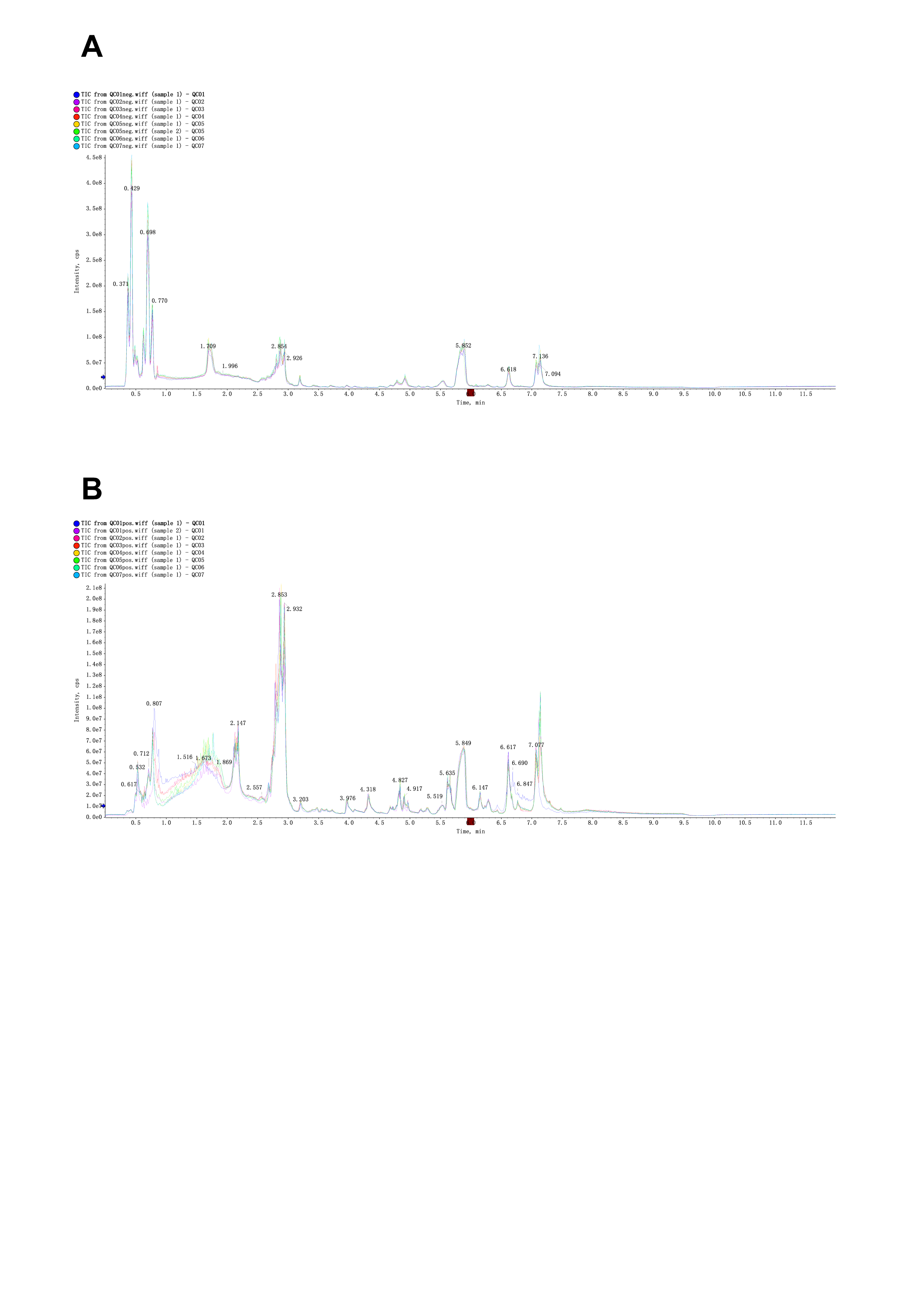

Supplement: Supplementary file 2 [file Image_1.TIF]

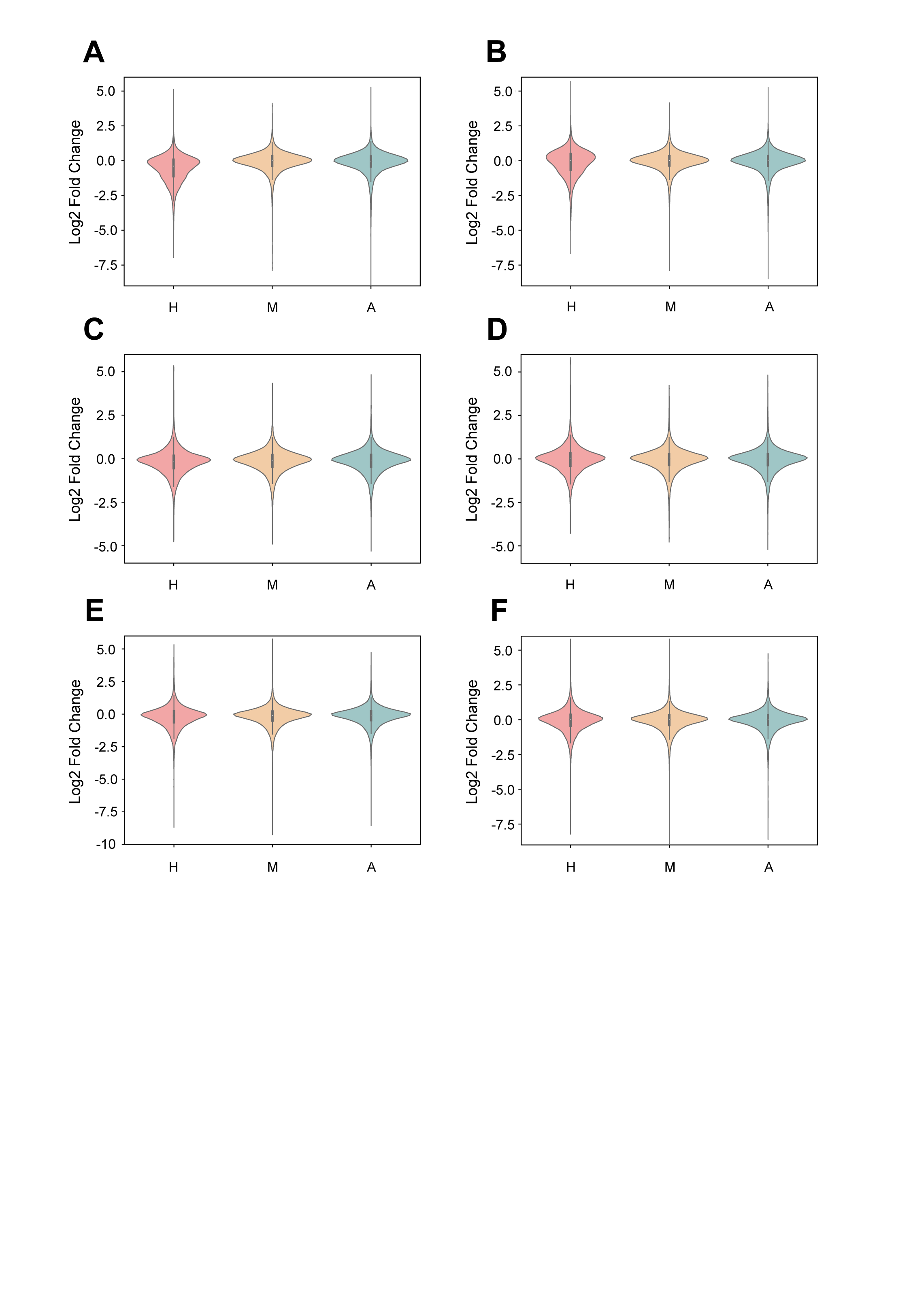

Supplement: Supplementary file 3 [file Image_2.TIF]

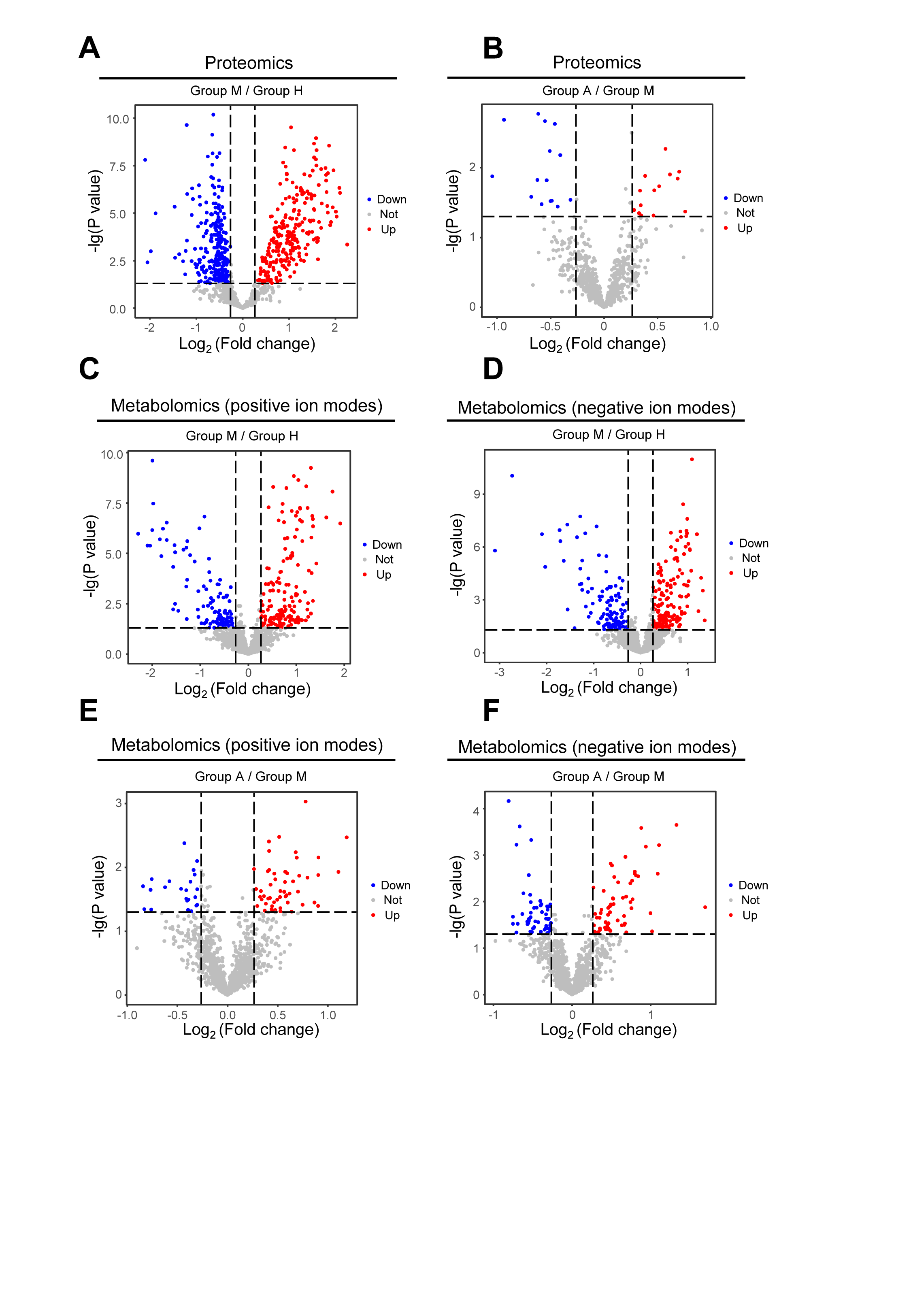

Supplement: Supplementary file 4 [file Image_3.TIF]

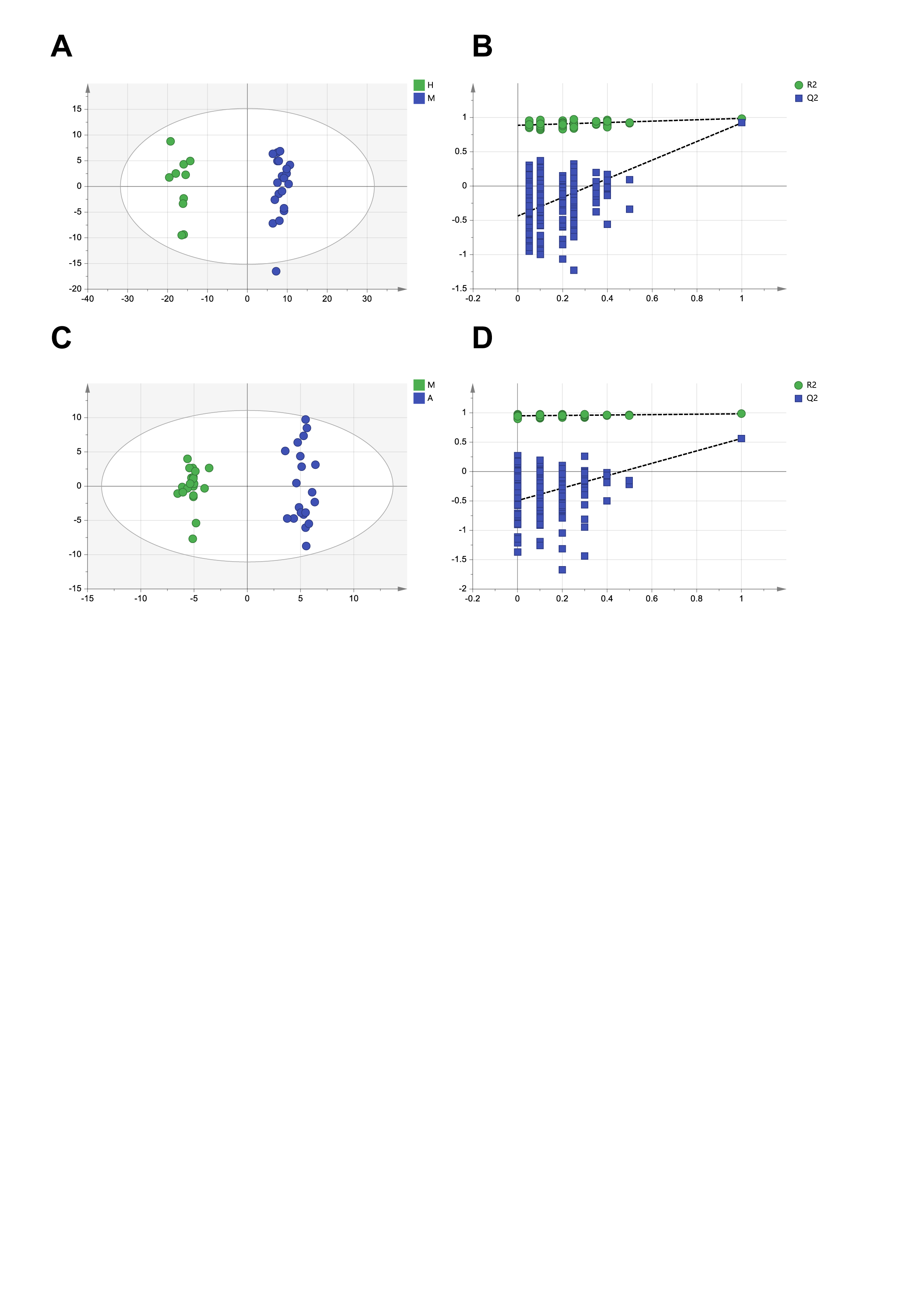

Supplement: Supplementary file 5 [file Image_4.TIF]

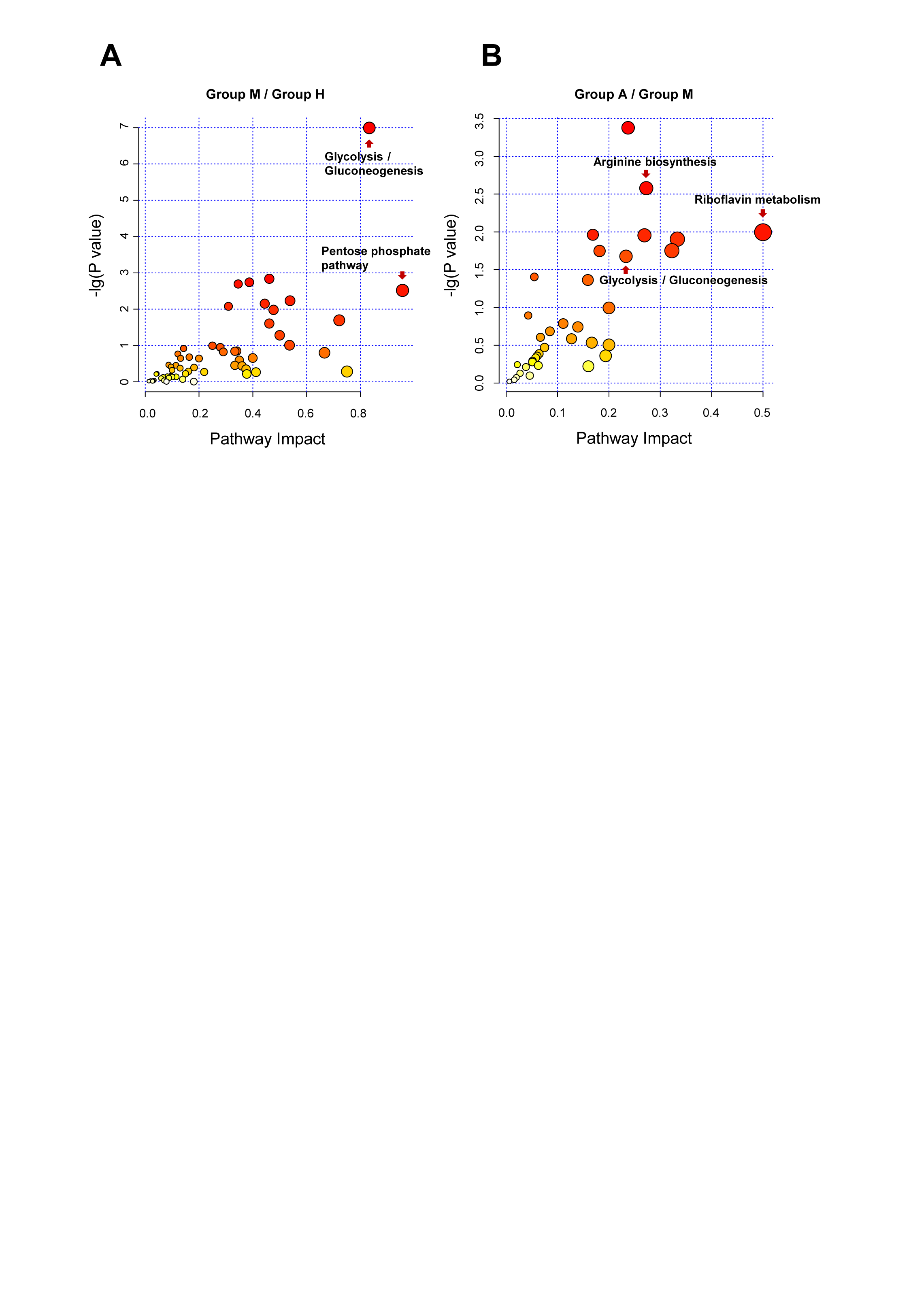

Supplement: Supplementary file 6 [file Image_5.TIF]

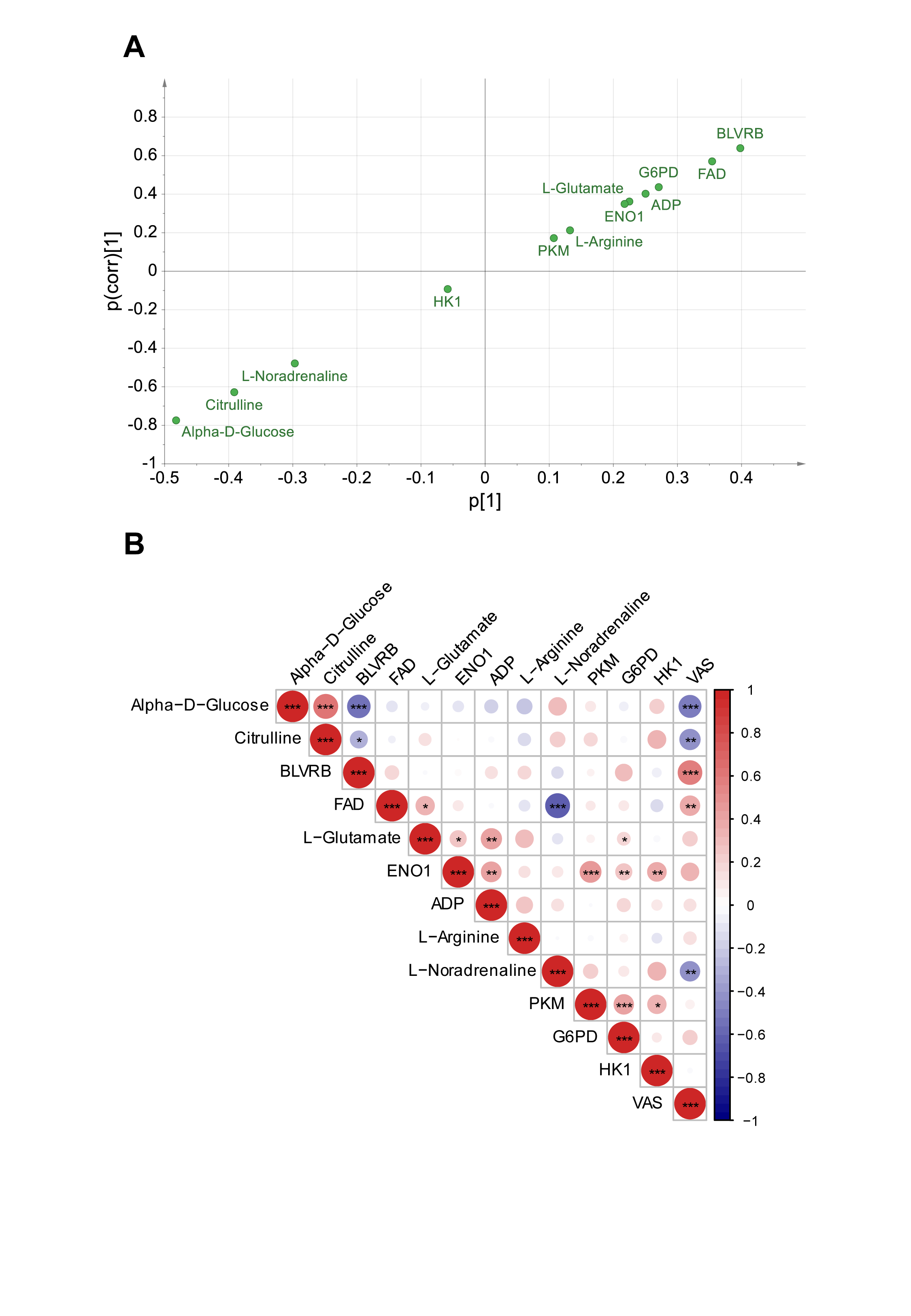

Supplement: Supplementary file 7 [file Image_6.TIF]

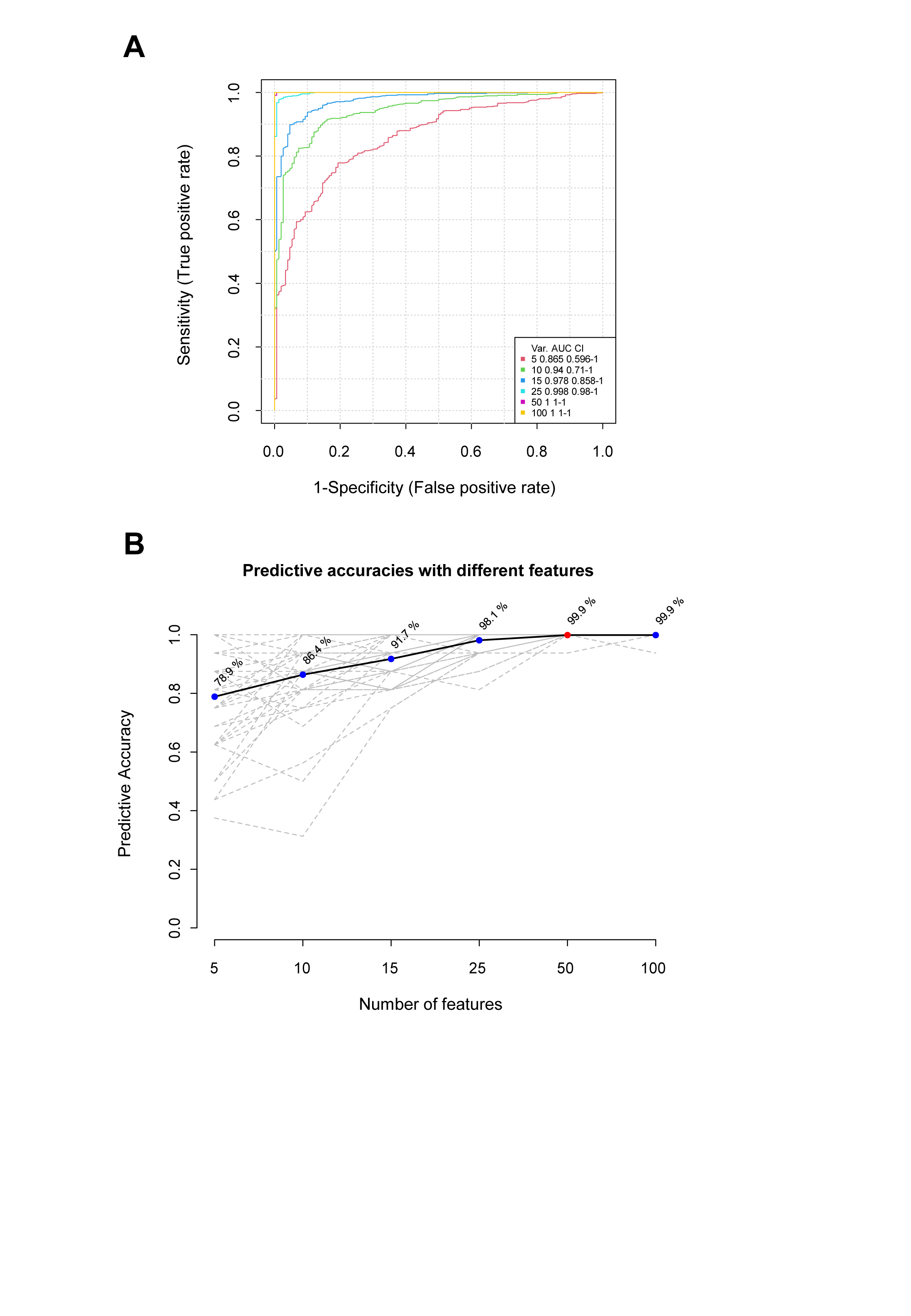

Supplement: Supplementary file 8 [file Image_7.TIF]
